# Polydatin Prevents Calcium Pyrophosphate Crystal-Induced Arthritis in Mice

**DOI:** 10.3390/nu13030929

**Published:** 2021-03-13

**Authors:** Francesca Oliviero, Paola Galozzi, Anna Scanu, Francesca Galuppini, Vanni Lazzarin, Silvia Brocco, Giampietro Ravagnan, Paolo Sfriso, Roberta Ramonda, Paolo Spinella, Leonardo Punzi, Gianmaria Pennelli, Roberto Luisetto

**Affiliations:** 1 Rheumatology Unit, Department of Medicine—DIMED, University of Padova, 35128 Padova, Italy; paola.galozzi@unipd.it (P.G.); anna.scanu@unipd.it (A.S.); paolo.sfriso@unipd.it (P.S.); roberta.ramonda@unipd.it (R.R.); 2 Surgical Pathology Unit, Department of Medicine—DIMED, University of Padova, 35128 Padova, Italy; francesca.galuppini@unipd.it (F.G.); vanni.lazzarin@unipd.it (V.L.); gianmaria.pennelli@unipd.it (G.P.); 3 Radiology Unit, Department of Medicine—DIMED, University of Padova, 35128 Padova, Italy; silviabrocco@yahoo.it; 4 Institute of Translational Pharmacology-National Research Council, 00133 Rome, Italy; gprav@unive.it; 5 Clinical Nutrition Unit, Department of Medicine—DIMED, University of Padova, 35128 Padova, Italy; paolo.spinella@unipd.it; 6 Centre for Gout and Metabolic Bone and Joint Diseases, Rheumatology, SS Giovanni and Paolo Hospital, 30122 Venice, Italy; punzileonardo@gmail.com; 7 Department of Surgery, Oncology and Gastroenterology-DISCOG, University of Padova, 35128 Padova, Italy; roberto.luisetto@unipd.it

**Keywords:** calcium pyrophosphate crystals, inflammation, polydatin, bioactive compounds, crystal-induced arthritis, prevention

## Abstract

Background: Polydatin is a stilbenoid with important antioxidant, anti-inflammatory, and immunomodulating properties. The aim of this study was to assess the anti-inflammatory preventive effect of polydatin in the mouse model of acute arthritis induced by calcium pyrophosphate (CPP) crystals. Methods: Acute arthritis was induced by the injection of a suspension of sterile CPP crystals into the ankle joint of Balb/c mice. Animals were randomized to receive polydatin or colchicine (the control drug) according to a prophylactic and a therapeutic protocol. The primary outcome was the variation of ankle swelling obtained after crystal injection and treatment, while histological parameters such as leukocyte infiltration, IL-1ß and CXCL1 levels and tissue expression were considered as secondary outcomes. Results: Prophylactic treatment with PD significantly diminished ankle swelling after 48 h from crystal injection. Secondary outcomes such as leukocyte infiltration, necrosis, edema, and synovitis were also decreased. PD caused a reduction in circulating levels of IL-1ß and CXCL1, as well as their tissue expression. By contrast, the therapeutic administration of PD did not have any beneficial effect. Conclusions: PD can effectively prevent acute inflammatory response to crystals in the mouse model of CPP crystal-induced arthritis. These results suggest that this bioactive compound might be used in the prevention of crystal-induced acute attacks in humans.

## 1. Introduction

Acute calcium pyrophosphate (CPP) crystal arthritis is caused by the deposition of CPP crystals in articular and periarticular tissues. It is characterized by the massive release of cytokines and other pro-inflammatory mediators at the site of inflammation and, clinically, by pain and limited joint function. Its acute onset and self-limiting painful synovitis led, originally, to classify the disease as “pseudogout” [1].

Indeed, gout and pseudogout share multiple common features; but, contrary to the precipitation of monosodium urate (MSU) crystals that can be prevented through the use of hypouricemic drugs, there is no pharmacological therapy that can prevent the formation of CPP crystals or promote their dissolution [2,3]. Moreover, CPP crystal formation derives from a complex and tightly regulated mechanism involving extracellular inorganic pyrophosphate and different enzymatic systems [4]. As a consequence, the pharmacological treatment of acute CPP crystal arthritis has been confined to anti-inflammatory drugs, colchicine, and corticosteroids administered both orally and by intra-articular injections [5].

Polydatin (PD), the natural glycoside precursor of resveratrol, is a stilbene that is mainly contained in grapes and the bark of *Polygonum cuspidate*. Several experimental models have demonstrated its antioxidant, anti-inflammatory, and immunomodulating properties [6,7,8,9]. We have recently shown that this compound is able to prevent the inflammatory response to pathogenic crystals in vitro [10]. The pretreatment of a monocytic cell line with PD before MSU and CPP crystal stimulation, in fact, showed to effectively block the production and expression of IL-1ß, which represents the most important driver for crystal-induced inflammation.

This study aimed at assessing the anti-inflammatory preventive effect of PD in the mouse model of acute CPP crystal-induced arthritis.

## 2. Materials and Methods

### 2.1. Mice

Male wild type, Balb/c mice of 10 weeks of age were bred and maintained under specific pathogen-free conditions at the animal facility of the Interdepartmental Research Center of the Experimental Surgery of Padova University.

All animal care and experimentation were conducted in compliance with the guidelines of the European Union Directive 2010/63 and the Italian Law D.Lgs. 26/2014 and with the approval of the Institutional Animal Experimentation Ethics Committee of Padova University and the Italian Health Ministry (Rome, Italy) registered under #102/2020-PR.

### 2.2. CPP Crystal-Induced Arthritis Development

Acute arthritis was induced by the injection of a suspension of 0.3 mg sterile CPP crystals (InvivoGen, Aurogene, Italy) in 20 μL PBS into the right ankle joint of the mice. Injections were performed under inhalant anesthesia (Sevorane^®^, Abbott, 4% induction, 1.5% maintenance) using a Fluovac respiratory system (Harvard Apparatus, Holliston, MA, USA) and microliter syringes #705 (Hamilton, Reno, USA) with 27 G beveled needles. Animals were randomized into 4 groups (n = 8 per group) receiving: (1) i.a. CPP crystals, (2) i.a. CPP crystals + PD, (3) i.a. CPP crystals + colchicine (control drug), (4) i.a. PBS (control group).

Ankle swelling was measured at different time points using a precision digital caliper (Kroeplin Gmbh, Schlüchtern, Germany). To avoid any attribution bias, ankle swelling was measured by an investigator who was blinded to the group allocation. Forty-eight hours after the injection of CPP crystals (peak of the acute phase) (preliminary experiments) mice were euthanized, and peripheral blood and ankle joints were collected for inflammatory cytokine assessment and histological analysis, respectively.

### 2.3. Drugs

PD was extracted from *Polygonum cuspidatum*, according to the procedure described in patent EP 1292320 B1; and kindly supplied by GLURES Srl (a spin-off of the National Research Council, Rome, Italy, purity > 99%). Colchicine was obtained from Sigma–Aldrich (Milan, Italy).

### 2.4. Treatment with PD and Colchicine

**Figure 1:**
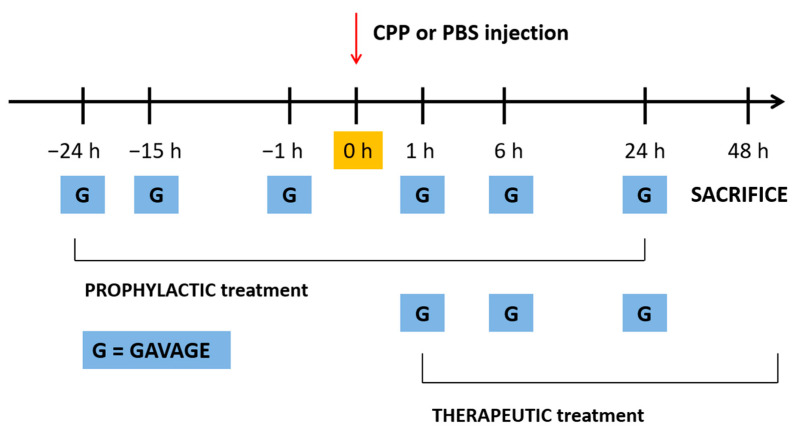
Prophylactic and therapeutic treatment protocols used in the mouse model of calcium pyrophosphate (CPP) crystal-induced arthritis. Gavage (G) time points are described in the figure for the two treatment protocols. In the prophylactic model, polydatin or colchicine have been administered at 24, 15 and 1 h before and 1, 6 and 24 h after crystal injection. In the therapeutic model, drugs have been administered 1, 6 and 24 h after crystal injection. Mice have been sacrificed 48 h after crystal injection.

Polydatin and colchicine were administered by gavage at 40 mg/kg and 1 mg/kg in 200 μL PBS/EtOH/glucose, respectively, according to two treatment protocols: 24, 15 and 1 h before and 1, 6 and 24 h after (prophylactic model) or 1, 6 and 24 h after (therapeutic model) i.a. injection of CPP crystals (Figure 1). These time points were chosen based on previous experimental data obtained by Reber and *colleagues* from an animal model with a similar joint involvement [11].

### 2.5. A Priori Sample Size Calculation, Primary and Secondary Outcomes

The effectiveness of PD administration in decreasing ankle swelling following CPP crystal injection was considered as the primary outcome of the study.

The sample size calculation was based on the expected change in ankle swelling between the control and the treatment group which was established as equal to 0.4 mm (variance intra-groups). According to the one-way ANOVA analysis, with statistical power and alpha levels set at, respectively, 0.8 and 0.05, the sample size resulted in 8 animals per group.

Leukocyte infiltrate and synovitis reduction, the decrease of local inflammation at ultrasound evaluation, serum, and tissue biomarkers reduction (IL-1ß, CXCL1) were used as secondary outcome measures.

### 2.6. Histological Assessment

Ankle joints were fixed in 10% buffered formalin and decalcified for 24 h using a solution of formic and nitric acid. They were then embedded in paraffin, cut into 4-μm-thick sections and stained with hematoxylin and eosin for evaluation. Slides were analyzed using a Leica DM4000B microscope provided with a Leica DFC420 camera.

A four-tier system (0–3) was used to assess leukocyte infiltration, necrosis and edema, and synovitis. The following scores were used: 0 =  normal, 1  =  mild effect, 2  =  moderate effect, 3  =  severe effect.

### 2.7. Ultrasound Assessment

Ultrasound assessment of the ankle joint was carried out in 2 mice per group in the prophylactic model. The exam was carried out by a radiologist, who was blinded to the mouse allocation group, using a Vevo 2100 machine (Fujifilm, Visualsonics, Toronto, ON, Canada) operating with the 22–55 MHz MS-550D MicroScan transducer. Tissues were observed in static (B-mode) and dynamic mode (M-mode). The following features were investigated: power Doppler signal, morphology, and hyperechogenicity of soft tissues.

### 2.8. RNA Extraction from Ankle Joint and RT-PCR

Total RNA from the mice’s ankle tissues was isolated according to the manufacturer’s instructions (Total RNA purification kit, Norgen Biotek Corp, Thorold, ON, Canada). RNA quality was examined using a NanoDrop Lite spectrophotometer (Thermo Fisher Scientific, Waltham, MA, USA), and then reverse-transcribed using an iScript™ Reverse Transcription Supermix (Bio-Rad, Milano, Italy) according to the manufacturer’s instructions. Amplification of IL-1β and CXCL1 genes was undertaken by an ABI Prism 7900HT (Applied Biosystems, Foster City, CA, USA), and were analyzed in duplicate for each sample. PCR reaction using iTaq Universal SYBR Green Supermix (BioRad, Hercules, CA, USA) was run at the following thermal cycling conditions: 95 °C for 30 s, followed by 40 cycles at 95 °C for 15 s and 60 °C for 1 min. Levels of mRNA for each target gene were normalized to 18S as reference gene and calculated according to the 2^−ΔΔCt^ method [12]. The sequences of primers used are as follow:

IL-1ß Forward: 5′-CGCAGCAGCACATCAACAAG-3′
Reverse: 5′-GTGCTCATGTCCTCATCCTG-3′
CXCL1 Forward: 5′-ATCCAGAGCTTGAAGGTGTTG-3′
Reverse: 5′-GTCTGTCTTCTTTCTCCGTTACTT-3′
18. S Forward: 5′ GGGAGCCTGAGAAACGGC 3′
Reverse: 5′ GGGTCGGGAGTGGGTAATTT 3′

### 2.9. Serum Cytokine Determination

Blood samples were collected by intracardiac puncture under general anesthesia and plasma obtained after centrifugation. Levels of IL-1ß and CXCL1 were measured by commercially available enzyme immunoassays (IL-1, eBioscience, sensitivity 8 pg/mL; CXCL1, Invitrogen, sensitivity 2 pg/mL).

### 2.10. Statistical Analyses

Data are expressed as mean ± standard deviation (SD). Analysis of variance (Kruskal–Wallis) followed by Dunn’s multiple comparison test was used to assess the effect of PD and colchicine in the prophylactic and therapeutic groups.

The Mann–Whitney test was used to compare the delta swelling between treatments at different time points.

## 3. Results

### 3.1. Prophylactic Oral Treatment with PD Prevents CPP Crystal-Induced Arthritis in Mice

**Figure 2:**
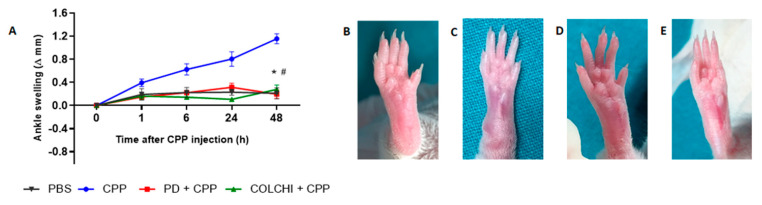
Variation in ankle swelling after CPP crystal injection and drug treatment in the prophylactic group. (**A**) Mice received PD (40 mg/kg) or colchicine (1 g/kg) treatment before the i.a. injection of CPP crystals (0.3 mg/20 μL) (n = 8/group). Analysis of variance by Kruskal–Wallis test for CPP group, *p* = 0.0025. Mann–Whitney test CPP group vs. PD + CPP group and colchicine + CPP at 48 h, * *p* < 0.01; CPP group vs. colchicine + CPP at 48 h, # *p* < 0.01. Delta swelling at each time point has been calculated with respect to the basal point. (**B**–**E**) Pictures depict ankles at the endpoint of the study (48 h): (**B**) PBS injection; (**C**) CPP injection; (**D**) CPP and prophylactic treatment with PD; (**E**) CPP and prophylactic treatment with colchicine.

CPP crystal injection in the ankle of the mice caused a progressive increase in joint swelling which was maximal at 48 h (preliminary experiments, not shown). This mean change was 1.15 ± 0.22 mm (Figure 2A). Prophylactic treatment with PD significantly reduced ankle swelling to 0.17 ± 0.115 mm after 48 h. This result was similar to that obtained in the group of mice treated with colchicine and injected with crystals according to the same model (mean delta ankle swelling 0.27 ± 0.15 mm).

**Figure 3:**
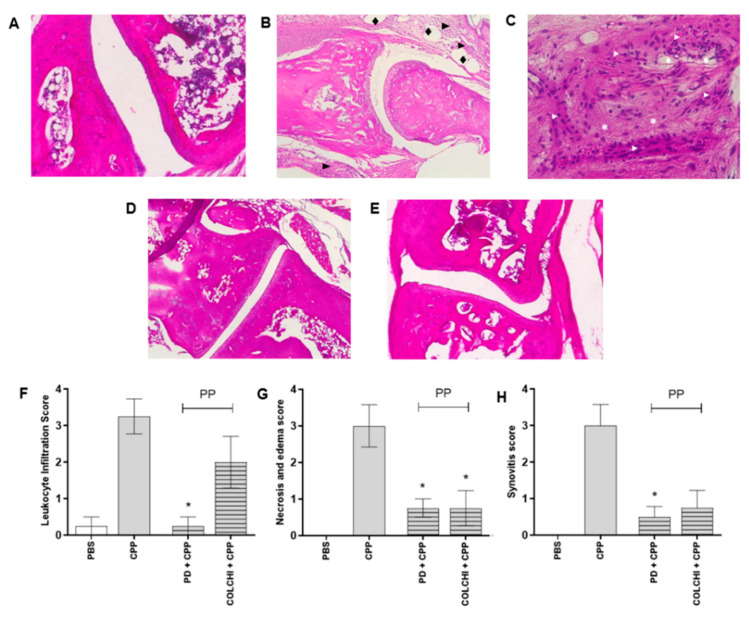
Leukocyte infiltration, necrosis and edema, and synovitis scores calculated on hematoxylin and eosin (H&E) stained sections of the ankles 48 h after CPP crystal injection. Mice were treated by gavage with PD (40 mg/kg) or colchicine (1 mg/kg) before and after (prophylactic) the injection of the crystals (0.3 mg/20 μL) (n = 8/group). Histological sections: (**A**) PBS 20 µL; (**B**) CPP; (**C**) detail of image B with symbols depicting inflammatory infiltrate (►), edema (♦) and necrosis (*); (**D**) PD + CPP (prophylactic); (**E**), colchicine + CPP (prophylactic). Magnification 10×. Histological parameters: (**F**) leukocyte infiltration, Kruskal–Wallis test *p* = 0.0019, * *p* < 0.05 vs. CPP; (**G**) necrosis and edema, Kruskal–Wallis test *p* = 0.0016, * *p* < 0.05 vs. CPP; (**H**), synovitis, Kruskal–Wallis test *p* = 0.0086, * *p* < 0.05 vs. CPP.

In mice treated with CPP crystals for 48 h, histological analysis revealed areas of edema and increased cell infiltrate in articular and periarticular tissues and the presence of reactive lymphnodes (Figure 3). Tissue necrosis around inflamed tissue was observed. Prophylactic treatment with PD importantly reduced cell infiltrate, necrosis and edema, and synovitis (Figure 3F–H). Although to a lesser extent, the same effect was obtained after the prophylactic administration of colchicine (Figure 3F–H).

**Figure 4:**
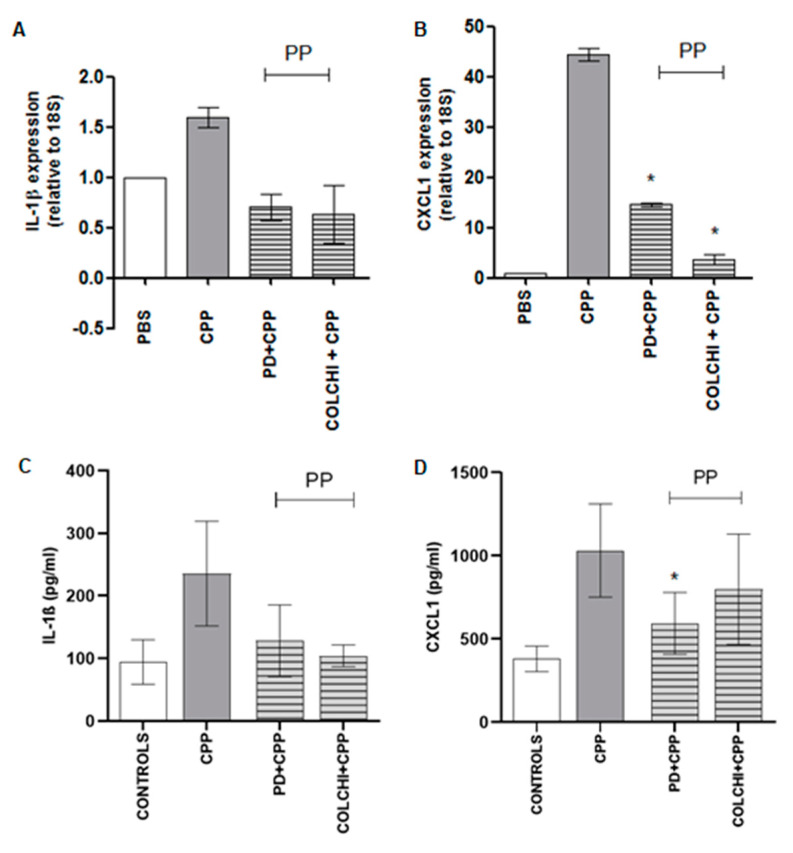
Variation in IL-1β and CXCL1 mRNA tissue levels (**A**,**B**) and serum levels (**C**,**D**) after CPP crystal injection in the prophylactic (PP) protocols. Mice received an i.a. injection of CPP crystals (0.3 mg/20 μL) and were treated with PD (40 mg/kg) or colchicine (1 g/kg) according to the group allocation (*n* = 8/group). Tissues were immediately stored in RNA-stabilizing reagent after sacrifice. * *p* < 0.05 vs. CPP. Cytokines were measured in blood collected by intracardiac puncture and after centrifugation. * *p* < 0.05 vs. CPP.

The gene expression study on ankle tissue revealed a 1.5- and 40-fold increased expression in IL-1ß and CXCL1 genes, respectively (Figure 4A,B). PD and colchicine administered prophylactically caused a non-significant reduction in IL-1ß and CXCL1 mRNA levels. As regards serum IL-1ß and CXCL1 levels, they increased significantly (*p* < 0.05) after 48 h from the injection of the crystals and showed a non-significant diminution after prophylactic treatment (Figure 4C,D).

### 3.2. Therapeutic Oral Treatment with PD Does Not Affect CPP Crystal-Induced Arthritis in Mice

**Figure 5:**
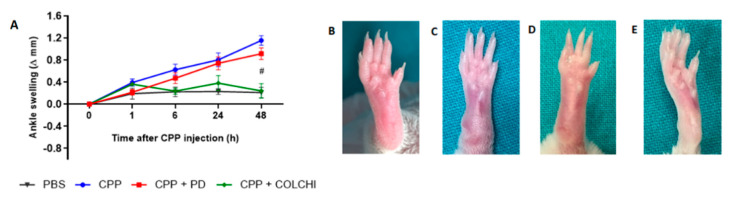
Variation in ankle swelling after CPP crystal injection and drug treatment in the therapeutic group. (**A**) Mice received PD (40 mg/kg) or colchicine (1 g/kg) treatment after the i.a. injection of CPP crystals (0.3 mg/20 μL) (*n* = 8/group). Mann–Whitney test CPP group vs. CPP + colchicine at 48 h, # *p* < 0.01. Delta swelling at each time point was calculated with respect to the basal point. (**B**–**E**) Pictures depict ankles at the endpoint of the study (48 h): (**B**) PBS injection; (**C**) CPP injection; (**D**) CPP and therapeutic treatment with PD; (**E**) CPP and therapeutic treatment with colchicine.

The therapeutic administration of PD did not have beneficial effects on delta swelling 48 h after CPP crystal injection (0.91 ± 0.25 mm) (Figure 5A). By contrast, colchicine significantly reduced ankle swelling in the therapeutic group of mice injected with the crystals (0.24 ± 0.29 mm). The effect of colchicine was, therefore, similar in both the protocols. Histological parameters did not show any improvement in the therapeutic group treated with PD except for synovitis score which showed a 2-fold non-significant decrease.

**Figure 6:**
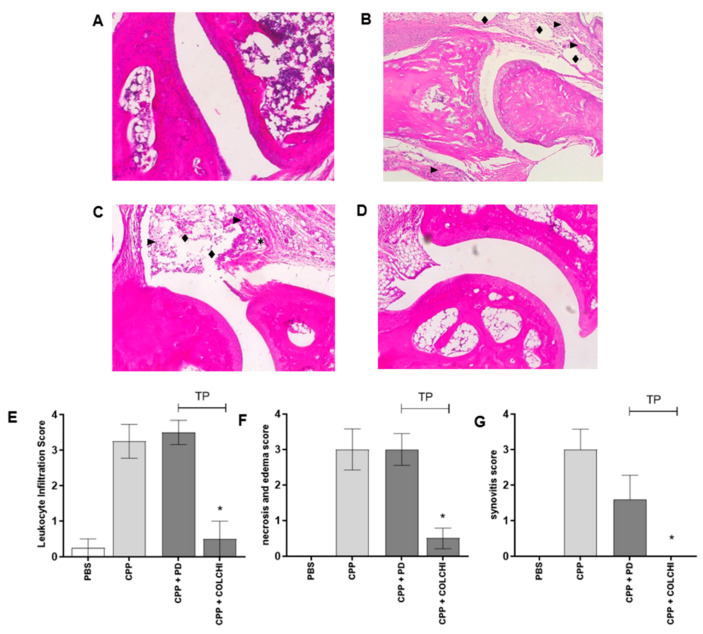
Leukocyte infiltration, necrosis and edema, and synovitis scores calculated on H&E-stained sections of the ankles 48 h after CPP crystal injection. Mice were treated by gavage with PD (40 mg/kg) or colchicine (1 mg/kg) after (therapeutic) the injection of the crystals (0.3 mg/20 μL) (n = 8/group). Histological sections: (**A**) PBS 20 µL; (**B**) CPP; (**C**) CPP + PD (therapeutic); (**D**) CPP + colchicine (therapeutic). Symbols depict inflammatory infiltrate (►), edema (♦) and necrosis (*). Magnification 10×. Histological parameters: (**E**) leukocyte infiltration, Kruskal–Wallis test *p* = 0.0019, * *p* < 0.05 vs. CPP; (**F**) necrosis and edema, Kruskal–Wallis test *p* = 0.0016, * *p* < 0.05 vs. CPP; (**G**) synovitis, Kruskal–Wallis test *p* = 0.0086, * *p* < 0.05 vs. CPP.

Leukocyte infiltration, necrosis and edema, and synovitis, all significantly decreased only in mice following the therapeutic treatment with colchicine (Figure 6).

**Figure 7:**
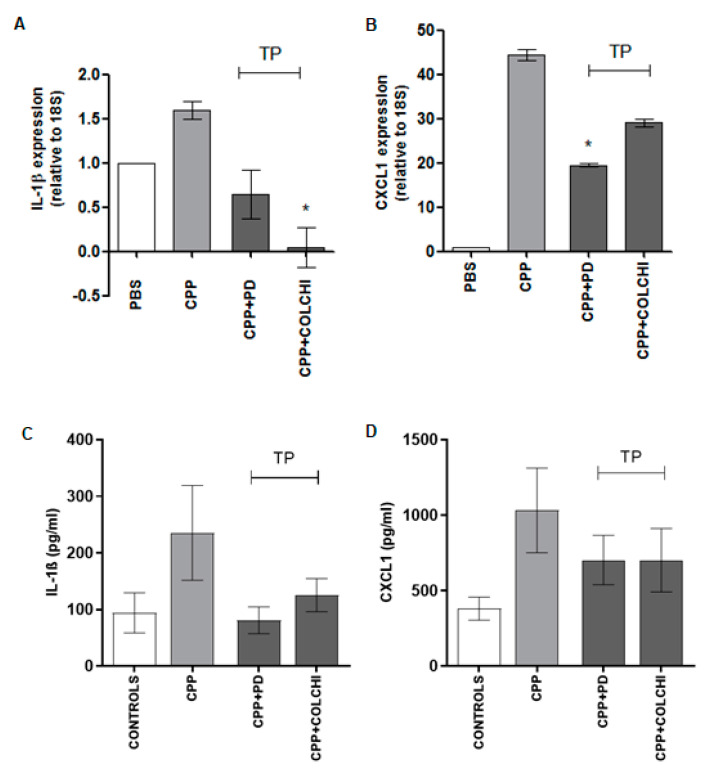
Variation in IL-1β and CXCL1 mRNA tissue levels (**A**,**B**) and serum levels (**C**,**D**) after CPP crystal injection in the therapeutic (TP) protocol. Mice received an i.a. injection of CPP crystals (0.3 mg/20 μL) and were treated with PD (40 mg/kg) or colchicine (1 g/kg) according to the group allocation (n = 8/group). Tissues were immediately stored in RNA-stabilizing reagent after sacrifice. * *p* < 0.05 vs. CPP. Cytokines were measured in blood collected by intracardiac puncture and after centrifugation. * *p* < 0.05 vs. CPP.

As far as cytokine expression was concerned, mice in the therapeutic protocol showed reduced IL-1ß mRNA levels when treated with PD and significantly lower levels when treated with colchicine (*p* < 0.05) (Figure 7A). The CXCL1 tissue expression decreased significantly after PD and non-significantly after colchicine therapeutic treatment. Serum levels of the same cytokines were also reduced in both treatment groups, although without reaching any significance (Figure 7C,D). While the results obtained in the group of mice treated with colchicine were in line with the clinical and histological observations, those evidenced in the therapeutic group of animals treated with PD were in contrast with the increase of swelling, cell infiltrate, and necrosis observed in this group (Figure 5 and Figure 6).

### 3.3. Ultrasound Evaluation of CPP Crystal-Induced Arthritis in Mice Treated Prophylactically with Polydatin

**Figure 8:**
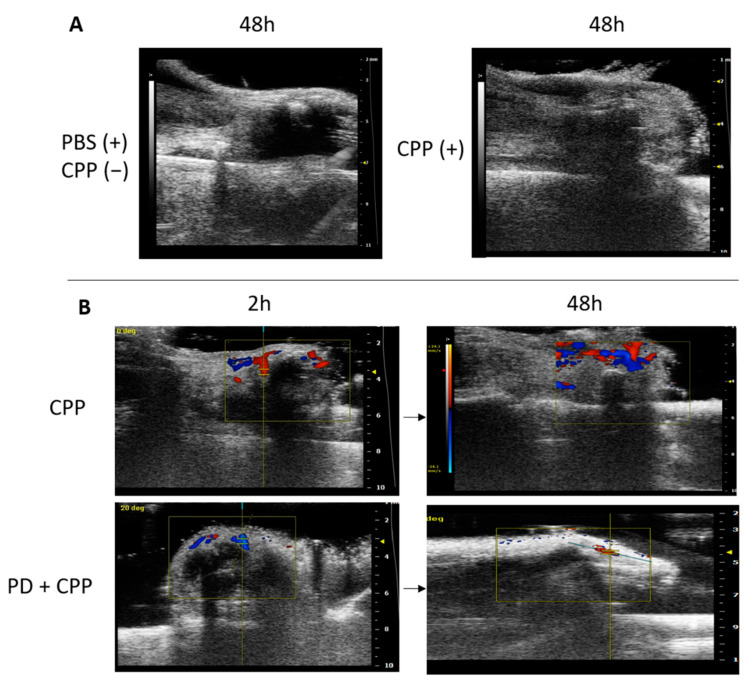
Ultrasound images of the right ankle joint obtained before and after CPP crystal injection and according to prophylactic treatment with polydatin. US performed on mice under anesthesia 48 h after PBS or CPP crystal (0.3 mg/mL) injection (**A**). Power Doppler US detection of hyperemia 2 h and 48 h after crystal injection (**B**, first line). Effect of prophylactic treatment with PD (40 mg/mL) on vascular changes caused by the crystals (**B**, second line).

The ultrasound (US) imaging study was performed to better evaluate the inflammatory signs in the group of mice injected with CPP crystals and treated with PD according to the prophylactic protocol. At the endpoint of the study, which corresponded to the acute phase of the disease, soft tissues showed a hyperechoic area in the site of crystal injection, when compared to the anechoic area caused by the PBS injection (Figure 8A).

Interestingly, the Doppler signal in the ultrasound images showed increased hyperemia after 2 h from the injection of the crystals, indicating an early inflammatory process which then increased during the acute phase (Figure 8B). As outlined in the figure, PD administered as prophylactic treatment limited the inflammatory reaction to crystals as evidenced by the reduced Doppler signal recorded after 48 h (Figure 8B).

## 4. Discussion

Our study demonstrated that PD can suppress CCP crystal-induced acute arthritis in mice. PD, administered according to a prophylactic protocol, effectively prevented ankle swelling after 48 h from crystal injection. As evidenced by the histological analysis, the secondary outcomes such as leukocyte infiltration, necrosis, edema, and synovitis were also diminished following the prophylactic treatment with PD. Polydatin caused a significant decrease in circulating levels of CXCL1, as well as its tissue expression; and, although non-significant, a reduction of IL-1ß mRNA and protein.

The effect of the treatment with PD was similar to that obtained with colchicine, the most common drug used to treat acute episodes of crystal-induced arthritis. Colchicine also remained effective in the therapeutic protocol; however, the treatment with PD did not lead to a favorable clinical change when administered therapeutically (i.e., after crystal injection).

Of note, variation in IL-1ß and CXCL1 protein levels and tissue expression in the therapeutic group did not differ from that observed in the prophylactic group. In the latter, we observed a moderate inhibition of both cytokines considered. This was expected for colchicine, which proved to be clinically effective in both protocols, but was slightly in contrast with the clinical evidences observed in mice treated therapeutically with PD. In this group, in fact, swelling increased after 48 h to levels comparable to the CPP-treated group. Results were also in contrast with the histological findings which showed pathological features and a worsening of the disease. Although we don’t have a clear explanation for this discrepancy, it is possible that PD administered after the injection of the crystals can’t rapidly hamper the inflammatory process triggered by the crystals themselves allowing clinical symptoms to reveal. The effect on cytokines observed after 48 h might consequently influence clinical outcomes at a later stage.

While additional studies are necessary to clarify this point, these results are in accordance with those obtained by our previous work [10]. Using an in vitro model of MSU and CPP-induced inflammation we demonstrated that the pre-treatment of THP-1 cells with PD abolished the production and mRNA levels of IL-1ß, and oxidative stress induced by the crystals without affecting crystal-phagocytosis, which is now recognized as an important step in the resolution of the acute inflammatory attack [13].

PD is the natural precursor of resveratrol. It is present predominantly in the roots and rhizomes of Polygonum cuspidatum but it can also be found in many vegetable foods and fruits, such as grapes [14]. With respect to resveratrol, PD is more resistant to enzymatic oxidation and it is soluble in water. Furthermore, its bioavailability is higher than that of resveratrol because it enters the cells via an active mechanism using glucose carriers [15].

The preventive anti-inflammatory properties of PD have been observed in different inflammatory experimental models of disease. It has been shown to prevent bleomycin-induced pulmonary fibrosis by inhibiting the TGF-β signaling pathway in rats [16]. Administered 1 h before treatment, PD has demonstrated to prevent lipopolysaccharide-induced acute kidney injury in mice. In this model, the polyphenol showed that it inhibited inflammatory and oxidative responses through, respectively, the suppression of the nuclear factor-κB (NF-κB) activation and myeloperoxidase activity, and the increase in the nuclear factor (erythroid-derived 2)-like 2 (Nrf2) and HO-1 expression [17]. The influence on NK-kB pathway is of particular interest given that it regulates IL-1ß mRNA transcription and secretion [18]. As this transcription factor, in turn, induces more IL-1, PD could exert anti-inflammatory actions by modulating the vicious cycle of sustained inflammation [19].

The inhibition of Nrf2 and pro-inflammatory cytokines has been also observed after stimulation of human osteoarthritic chondrocytes stimulated with IL-1β and treated with PD [20]. In the surgical model of osteoarthritis obtained through the destabilization of the medial meniscus, PD was shown to alleviate disease progression through a reduction of typical signs of osteoarthritis such as joint space narrowing and synovitis [20]. With a similar anti-inflammatory mechanism, PD administration significantly improved myocardial dysfunction in diabetic rats, by inhibiting cytokine production and ROS [21]. A role in preventing liver inflammation and nonalcoholic fatty liver disease has also been postulated [22,23], as well as the regulation of the inflammatory state in adipose tissue [24].

Polydatin has shown to reach the brain and have neuroprotecting effects. It has been demonstrated to prevent the induction of secondary brain injury after trauma by exerting protective effects on neuronal mitochondria [25]. The inhibition of mitochondrial apoptotic pathways consequent to the increased expression and activity of sirtuin 1 (SIRT1) has been associated with this beneficial effect.

Overall, the studies focusing on PD underline its strong effect on inflammatory and oxidative pathways. However, the role of this bioactive compound has never been considered in crystal-induced arthritis. It has been demonstrated that its metabolite, resveratrol, inhibited MSU crystal-induced inflammation [26] and suppressed the onset of gout in mice by acting, in particular, on SIRT1 expression, and consequently on the levels of PPARγ [27]. Patients with gout have reduced levels of SIRT1, which might be, therefore, restored by resveratrol [26].

CPP crystal-induced arthritis shares common features with gout, in particular the abrupt onset with signs and symptoms of severe acute inflammation. But unlike gout, initial episodes of acute CPP crystal arthritis may persist longer before remitting [1,3].

In the animal, this might be reflected by the induction of the acute attack which developed after 24 h and resolved completely in 120 h from the injection of MSU crystals [28]. By contrast, after 144 h from CPP crystal injection, mice still presented swelling, redness, and edema which were only half-reduced with respect to the peak of the acute process (data not shown). Indeed, CPP crystals do not dissolve like MSU whose precipitation and dissolution depends on uric acid levels and other environmental factors. Once deposited in cartilage and soft tissues, CPP crystals cannot be safely dissolved and can lead to a chronic state of the disease [29].

In this view, PD might provide a potential benefit in different ways: preventing the onset of acute attacks, as evidenced in this study, and preventing the development of disease flares which can lead to the more difficult to treat chronic phase of the disease.

Our study has indeed some limitations. We cannot provide detailed information about the mechanisms of action behind the preventive effects of this polyphenol. From both our in vitro and in vivo experiments, we have substantial evidences that PD blocks the IL-1ß pathway before priming but it has no clinical effect, at least in the early stage, once the pathway is activated. It is likely that the pretreatment might condition the basal state of cells, making them less prone to stimulation. The absence of benefit in the therapeutic group of mice supports this hypothesis.

A strength of our study is the first-time description of the acute model of CPP crystal-induced arthritis. Reproducing the human acute attack, this model allows the study of inflammatory and structural joint changes induced by the crystals, and to test new pharmacological strategies in the management of this form of arthritis. Ultrasonography coupled with power Doppler analysis may, furthermore, point out early morpho-structural and synovial vascularity changes over time.

## 5. Conclusions

In summary, our study showed, for the first time, the beneficial effects of PD in preventing the acute articular inflammatory process induced by CPP crystals in mice and the consequent damage triggered by the crystals themselves. Although additional studies are needed to establish the long-term health effect of PD, our results suggest that this bioactive compound might be used as a dietary supplement in the prevention of crystal-induced acute attacks in humans.

## Data Availability

The data presented in this study are available on request from the corresponding author.
